# Evaluation of different machine learning algorithms for predicting the length of stay in the emergency departments: a single-centre study

**DOI:** 10.3389/fdgth.2023.1323849

**Published:** 2024-01-08

**Authors:** Carlo Ricciardi, Marta Rosaria Marino, Teresa Angela Trunfio, Massimo Majolo, Maria Romano, Francesco Amato, Giovanni Improta

**Affiliations:** ^1^ Department of Electrical Engineering and Information Technology, University of Naples “Federico II”, Naples, Italy; ^2^ Department of Public Health, University of Naples “Federico II”, Naples, Italy; ^3^ Department of Advanced Biomedical Sciences, University of Naples “Federico II”, Naples, Italy; ^4^ Interdepartmental Center for Research in Healthcare Management and Innovation in Healthcare (CIRMIS), University of Naples “Federico II”, Naples, Italy

**Keywords:** crowding, emergency department, length of stay, machine learning, classification algorithm

## Abstract

**Background:**

Recently, crowding in emergency departments (EDs) has become a recognised critical factor impacting global public healthcare, resulting from both the rising supply/demand mismatch in medical services and the paucity of hospital beds available in inpatients units and EDs. The length of stay in the ED (ED-LOS) has been found to be a significant indicator of ED bottlenecks. The time a patient spends in the ED is quantified by measuring the ED-LOS, which can be influenced by inefficient care processes and results in increased mortality and health expenditure. Therefore, it is critical to understand the major factors influencing the ED-LOS through forecasting tools enabling early improvements.

**Methods:**

The purpose of this work is to use a limited set of features impacting ED-LOS, both related to patient characteristics and to ED workflow, to predict it. Different factors were chosen (age, gender, triage level, time of admission, arrival mode) and analysed. Then, machine learning (ML) algorithms were employed to foresee ED-LOS. ML procedures were implemented taking into consideration a dataset of patients obtained from the ED database of the “San Giovanni di Dio e Ruggi d’Aragona” University Hospital (Salerno, Italy) from the period 2014–2019.

**Results:**

For the years considered, 496,172 admissions were evaluated and 143,641 of them (28.9%) revealed a prolonged ED-LOS. Considering the complete data (48.1% female vs. 51.9% male), 51.7% patients with prolonged ED-LOS were male and 47.3% were female. Regarding the age groups, the patients that were most affected by prolonged ED-LOS were over 64 years. The evaluation metrics of Random Forest algorithm proved to be the best; indeed, it achieved the highest accuracy (74.8%), precision (72.8%), and recall (74.8%) in predicting ED-LOS.

**Conclusions:**

Different variables, referring to patients’ personal and clinical attributes and to the ED process, have a direct impact on the value of ED-LOS. The suggested prediction model has encouraging results; thus, it may be applied to anticipate and manage ED-LOS, preventing crowding and optimising effectiveness and efficiency of the ED.

## 1 Introduction

Emergency departments (EDs) offer a fundamental service, providing emergency help 24 h a day, 365 days a year, therefore representing a critical public service role. The service is ensured by the presence of doctors assisting patients’ injuries and illnesses, both acute and chronic. The ability and expectation of providing fast and decisive access for patients experiencing a medical emergency are among the evaluation criteria of an ED’s performance (1).

There are two types of performance indicators: general indicators and specific ones. In particular, visit priority code and process time for assigned code are general indicators, while the relationship between length of hospital stay (LOS) and number of accesses for the code, the relationship between triage-visit time and number of accesses per code, the analysis of the flow of turnout in triage by time slots, and the application of the Pareto Rule for analysis of the performance of medical staff are specific indicators (2). Crowding in EDs has been recognised as a critical challenge in hospital administration (3, 4). ED crowding has been linked to a variety of negative consequences, including increased costs for admissions, longer duration of stays and waits, and higher mortality rates (5–8). Specifically, prolonged LOS is a significant evaluation metric for tracking ED crowding and its consequent saturation, which has been proved to be difficult to assess directly. As mentioned previously, LOS is identified as a marker of the quality of care in numerous healthcare settings and, as earlier research has shown, multiple aspects mainly associated with the patients’ characteristics and the healthcare workflow relate to its modification (9, 10).

The LOS in ED (ED-LOS) is defined as the interval from ED arrival to ED departure (11), meaning the period that passes between the registration of patients in the ED and their discharge from the ED, whether they are hospitalised in a medical ward, shifted to another centre, or discharged home (12). Having the capacity to recognise the variables that are significantly linked to high ED-LOS may be important since the health policy could use it to decrease the occurrence of this issue. Of course, a prolonged ED-LOS is a consequence of the crowding in EDs.

The LOS in healthcare can be influenced by a variety of circumstances and conditions. As a result, several methodologies have been proposed in the literature to study the factors that influence LOS in healthcare processes. Among them, regression models and artificial intelligence techniques have been widely applied with satisfactory performance to predict the LOS (13–19) and to address healthcare-related problems, such as elaboration and analysis of biomedical data and signals (20–26), development of clinical decision-making support systems (27, 28), and quality assessment of medicine services. In fact, LOS has been already employed as a target output in healthcare, and other studies have recently aimed at predicting it in different fields (29, 30).

Multiple studies show that ED-LOS is impacted by a multitude of complex variables, including hospital organisation and management, clinical staffing, bed occupancy, and triage procedure (31–34). The clinical status of patients, as well as information such as sex, age, and comorbidity, are all linked to ED-LOS (35).

A flow of patients via ambulance service exacerbates the problem by bringing additional patients into a department that is already at or near capacity. In the meantime, the clinical condition of already triaged subjects in the waiting room might worsen within minutes from the first registration, resulting in bad outcomes (36). Extended LOS also leads to provider discontent and subsequent burnout (37).

Actually, no precise cut-off has been set up for defining an extended ED-LOS, with estimates varying from 3 to 12 h (38, 39). Several studies have defined an extended ED-LOS as a period spent by patients waiting in the ED of more than 3 h and other studies showed that complications can occur in patients diagnosed after 3 h from ED admission (40–44). Furthermore, longer ED-LOS has a damaging effect on key areas including patient experience with emergency medical care, the risk of side effects, and the percentage of patients who abandon the ED without being seen (45).

As a result, the ability to anticipate LOS and minimise ED congestion by recognising features that have an impact on prolonged LOS is critical to improving emergency quality-of-care and assisting hospital managers in ED operational processes planning (42).

Machine learning (ML) techniques are frequently used in the health industry for supporting in diagnosis, forecasting patient outcomes, and allocating staff resources (46, 47). Following this approach, many researchers have examined ways to determine ED-LOS, exploiting data processing, and particularly ML strategies (48–50). ML allows employing several predictors (also known as features) to build models that are useful to classify an output (usually categorical variables in the context of classification). Differently, when the output is a numeric variable, regression algorithms are required; nevertheless, there are different algorithms that can be used for both classification and regression tasks.

Based on the aforementioned considerations, the aim of this work, which is an extension and improvement of a prior work presented at a conference (51), is to build an advantageous model to forecast ED-LOS in advance by applying ML classification techniques. Predicting the ED-LOS in advance, based on a few variables, would be beneficial in this case to plan for future adjustments in financial and staffing allocation with the goal of minimising ED-LOS.

## 2 Methods

### 2.1 Context

In this work, we analysed a large dataset of patients extracted from the ED database of the University Hospital of Salerno, “San Giovanni di Dio e Ruggi d'Aragona.” It is a University Hospital of national importance with the highest number of accesses in Campania region (an average of 95,000 accesses per year, with about 250 registrations per day at the ED, with only a part of them needing successive hospitalisation).

Table 1 shows the staff working at the ED and the availability of beds in the ED in this University Hospital, according to the admission code.

**Table 1:** Staff and beds available per triage code in the ED.

Staff	Available units
Medical managers	15
Nurses	80
Health and social operators	49
Technician	1
Administrative personnel	2
Auxiliary specialised personnel	3
Triage codes	Beds available
Green	Six medical Four surgical
Yellow	12
Red	4
Brief intensive observation	12

Moreover, there are 895 beds for acute patients.

### 2.2 Data collection

The collected data, analysed in this study, belong to the period between 2014 and 2019, with 496,172 admissions, to avoid the overlapping with the COVID-19 pandemic.

The dataset was prepared to make it conform with the ML algorithms processing. Specifically, in the analysis, we did not take into consideration all the situations in which the predictor variables or the ED-LOS were not present. This choice is justified by the fact that only 50 entries did not have all the features available and hence we deemed that the elimination of these 50 records would not alter the results of the model. The characteristics considered for each record of the dataset are as follows:

• Gender: male/female (coded as: 0/1 for further processing).
• Age: divided into four classes: under 19, 10–40, 41–64, over 64.
• Access mode: divided into two classes: (1) autonomous, which considers patients reaching the ED by themselves; (2) via ambulance, including patients accessing the ED by ambulance.
• Triage score: divided into five classes according to the colour assigned at the time of admission based on the severity of the patient's clinical condition, gradually increasing from white code (absence of severe symptoms) to green, yellow, red, and black code (death).
• Time of admission: split into the following time windows: 0:00–6:00, 06:00–12:00, 12:00–18:00, 18:00–24:00.

Among the features available in the considered ED dataset, the selection was mainly driven by the knowledge of the specialists. Physicians who experienced prolonged ED-LOS helped us divide the features according to their effects on LOS. Factors considered as having low impacts on LOS, i.e., nationality, residence, triage doctor on duty, were eliminated. Moreover, these input features for prediction are the variables reported as factors influencing LOS in the analysed literature. The characteristics considered to have high impacts on LOS were evaluated for each record of the dataset and are provided in Table 2. Of note, the chosen variables, which will then be used for building models, are easily available in all healthcare facilities, which means that the proposed process of analysis could be easily implemented.

**Table 2:** Dataset characteristics.

Feature	Type	Total	Percentage
Gender	M	257,174	51.8
	F	238,998	48.2
Triage Score	White	21,915	4.4
	Green	409,528	82.5
	Yellow	58,688	11.8
	Red	5,952	1.1
	Black	89	0.1
Arrival mode	Autonomous	410,078	82.6
	Via ambulance	86,094	17.4
Time of admission	0:00–6:00	38,982	7.9
	06:00–12:00	180,044	36.3
	12:00–18:00	181,674	36.6
	18:00–24:00	95,472	19.2

As requested by the health direction of the hospital, ED-LOS has been considered prolonged when it had a value greater than 3 h. The choice has been made also by considering distribution of the data, which did not allow us to consider a different threshold (which would have affected the creation of the classes). Moreover, in the literature, the most common thresholds are 2 and 4 h; therefore, a choice of 3 h could be acceptable (52, 53).

The dataset was characterised by 143,641 occurrences (28.9%) of LOS with more than 3 h, and 352,531 (71.1%) of LOS less than 3 h.

### 2.3 Machine learning

ML techniques work by learning a function that translates input data to an output to make a prediction of its value. This is a generic learning activity that helps in making future predictions based on new given samples of the same input parameters. In this study, ML classification algorithms were implemented to forecast the ED-LOS. To handle the data, the Colab platform, a Cloud computing platform that supports Python as a computer language, has been used to develop a script that, starting from the input parameters, automatically predicts the future trend of ED-LOS. In our analysis, sex, age, arrival mode, triage score, and admission time slot were employed as input data for the classification algorithms. As an output, the ED-LOS was converted into a categorical variable. The total ED-LOS was dichotomised using a cut-off value of 3 h, indicating a prolonged stay, as requested by the health direction of the hospital and necessary to obtain a dataset distribution that was not too imbalanced. Since we had a dataset with labelled classes with the ED-LOS for each patient, supervised learning algorithms were exploited. Four distinct algorithms were used for the classification: Random Forest (RF), Neural Network based on a Multilayer Perceptron (MLP), Naïve Bayes (NB), and Logistic Regression (LR). RF is focused on a method that involves bootstrapping to train numerous decision trees concurrently on different subgroups of the whole dataset and the given features. Next, using bagging, RF combines the outputs of the individual trees. RF has been chosen in our study because it is not sensitive to dataset noise, and it is not affected by overfitting; moreover, it works quickly and outperforms plenty of other tree-based methods. NB is a classification algorithm based on the principle of probability, specifically the NB theorem; it requires a robust constraint of feature independence although it consents to achieve good results in binary classification. Following the NB theoretical principles, the target of using this ML algorithm in our case study is to find what class of LOS has the maximum probability to occur based on the patient features. MLP is a classifier based on back propagation and refers to a multilayer feed-forward neural network mapping input data to output by estimating the weights associated with the network during training. Our analysis could have taken advantage of the MLP properties to be static, as for a given input they generate only one output set, and to be memoryless, because of the independence of the output from the previous network state. LR is a probabilistic clustering algorithm that is used to estimate probabilities that an input belongs to a given class using a logistic sigmoid function to allocate the forecast result to a class based on whether the probability is near the class itself. LR is perfectly suited to our study because predictions are made according to the presence or absence of characteristics (normal or prolonged LOS) based on a set of predictor variables. The decision to use these ML techniques was primarily motivated by the willingness to use algorithms following different theoretical approaches and, second, to improve the efficiency of the operations of learning on the dataset through a tuning of their parameters. The classifiers were all taken from the Apache Software Foundation's MLlib library, which is an ML library.

Because this is a single-centre study, an external validation has not be performed, but the performance of the algorithms was tested by using a ten-fold cross-validation to ensure that the accuracy value was more reliable than by using a hold-out validation. Moreover, a careful adjustment of the parameters of the classifiers was conducted depending on the individual properties of each, and the quality of the produced model was assessed.

When the four classifiers have outputted their predictions, a voter ensemble algorithm used their output to determine the majority class to be ascribed to the ED-LOS of the patient, meaning that, to get a greater performance, the voter employs an ensemble approach that relies on majority policy. Indeed, voting assigns to each record (i.e., patient) the value foreseen by at least three of the classifiers, resulting in a prediction matching to the option that obtains more than half of the votes.

The performance of the models was assessed by computing the following evaluation metrics:

• Accuracy represents the ratio of correct predictions over the total.
  $$\mathrm{Accuracy}=\;\frac{\mathrm{TP}+\mathrm{TN}}{\mathrm{TN}+\mathrm{FP}+\mathrm{FN}+\mathrm{TP}}$$
• F-measure is the harmonic mean between precision and recall.
  $$F\text{-}\mathrm{measure}=\;\frac{2\mathrm{TP}}{2\mathrm{TP}+\mathrm{FP}+\;\mathrm{FN}}$$
• Precision is the ratio between the amount of correct predictions of class over the total number of times the model predicts it.
  $$\mathrm{Precision}=\frac{\mathrm{TP}}{\mathrm{TP}+\mathrm{FP}}$$
• Recall is the ratio of correct predictions for a class over the total number of cases in which it actually occurs.
  $$\mathrm{Recall}=\frac{\mathrm{TP}}{\mathrm{TP}+\mathrm{FN}}$$

where TN is true negative, TP is true positive, FN is false negative, and FP is false positive. Finally, the 95% confidence interval was determined for each of the metrics using the Normal Approximation Interval method based on a test set (54).

## 3 Results

First, we present in a graphical way some characteristics of the patients enrolled in this study during the six years’ time analysed; 496,172 patients met the eligibility criteria and 143,641 of them (28.9%) revealed an ED-LOS greater than 3 h. Considering the sample selected, 238,998 females (48.1%) and 257,174 males (51.9%) were registered at the ED.

**Figure 1:**
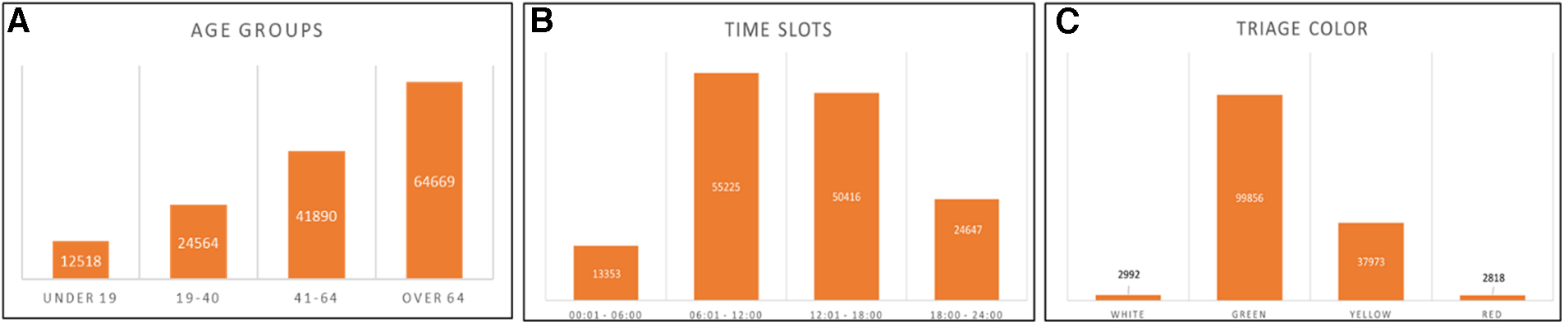
(**A**) Age group distribution for patients with prolonged ED-LOS. (**B**) Admission time slots for patients with prolonged ED-LOS. (**C**) Triage colour for patients with prolonged ED-LOS.

Among the patients with a prolonged LOS, 47.3% were female and 51.7% were male; 71.0% reached ED autonomously and the remaining, by ambulance. A histogram in Figure 1A shows the distribution of the ages among patients with prolonged ED-LOS:

Patients with higher ED-LOS belong mainly to the over 64 population.

Analysing then the time slots during which patients are admitted, it was determined that prolonged LOS are primarily present for the slot 06:00–18:00, with the ED being more frequented in these hours, as shown in Figure 1B.

Based on the assigned triage colour, it can be concluded that patients with the green code are the most numerous (Figure 1C) and, as it is reasonable to be, they have higher rates of LOS.

Second, the evaluation metrics of the four considered ML algorithms have been evaluated with the aim of investigating which best predicts the ED-LOS. Starting from the processing implemented through the Colab platform, four different performance measures (accuracy, F-measure, precision, and recall) have been calculated. The obtained values for the classification of ED-LOS are reported in Table 3.

**Table 3:** Evaluation metrics, expressed in %, and confidence intervals of the ML algorithms.

Algorithms	Accuracy	F-measure	Precision	Recall
RF	74.88	71.70	72.85	74.88
	(0.746–0.751)	(0.714–0.720)	(0.726–0.731)	(0.746–0.751)
MLP	74.69	72.13	72.59	74.69
	(0.744–0.750)	(0.718–0.724)	(0.723–0.729)	(0.744–0.759)
NB	71.75	62.33	68.43	71.75
	(0.715–0.720)	(0.620–0.626)	(0.681–0.687)	(0.715–0.720)
LR	74.33	69.86	72.36	74.33
	(0.741–0.746)	(0.695–0.701)	(0.720–0.726)	(0.740–0.746)
Voter	74.87	71.58	72.84	74.87
	(0.746–0.751)	(0.713–0.719)	(0.726–0.731)	(0.746–0.751)

The summarised results show that the accuracy reached by each classifier is comparable and the ensemble learning approach, which has been used to improve the performance, does not achieve a better score when compared with the other algorithms. Indeed, RF achieves the highest accuracy (74.88%), precision (72.85%), and recall (74.88%).

## 4 Discussion and conclusion

ED-LOS represents a crucial key indicator of the efficiency and appropriateness of healthcare services. The ability to understand the reasons of prolonged LOS in ED could reasonably support the detection of “bottlenecks” in their organisation. Indeed, the LOS is currently an important indicator for health facilities; other studies have already been focused on the use of such indicators as variables to be predicted for improving the efficiency of the hospital management (13–19).

Patient-related data, such as age, gender, admission time, and triage score, were included as predictors and analysed. The study was conducted involving a dataset of patients registered during the years 2014–2019 to the “San Giovanni di Dio e Ruggi d'Aragona” University Hospital's ED (Salerno, Italy). After collecting and analysing all data, we built a modelling approach to predict ED-LOS using the following ML algorithms: RF, MLP, NB, and LR. With the aim of developing a classification algorithm starting from sample or “training” data, the power of ML techniques was exploited. We extended the previous paper presented at a conference (51) by increasing the size of the dataset to train our prediction model, considering a longer timeframe and therefore a higher number of total entries. We improved the previous analysis considering ML algorithms that exploit different theoretical principles from each other, to evaluate their performance. Indeed, differently from other studies, the utilisation of a large volume of data offered us the possibility to train different sophisticated ML algorithms in an appropriate manner. The performance of the resulting prediction model is then evaluated to prove its capacity to enhance ED management.

The available data indicate that patients who exceed in ED-LOS belong to the elderly patients’ group (over 64) and that major issues are found in the time window between 6 a.m. and 6 p.m., when ED crowding may occur. Moreover, the results of this study revealed that the highest proportion from the sample representing the 80% of ED accesses is triaged as green code, i.e., non-urgent cases. This may be due to the lack of accessibility of primary healthcare facilities, insufficient availability to outpatient treatments, a poor understanding of the function of the ED, and inadequate discharged follow-up planning. Starting from patient's features and by means of ML, it is possible to pre-emptively detect extended ED-LOS. The consistency of the sample size employed for training the ML classifiers as well as the good value of the performance measures are two main advantages of our prediction model. We gathered a total of 496,172 entries, detecting 143,641 patients with prolonged ED-LOS (greater than 3 h), even after cleaning the dataset by eliminating all the records pulled from the University Hospital's database with missing details.

Lastly, the ensemble learning approach has been used to improve the performance of the data processing. To evaluate the voter's performance, the total value of accuracy achieved is determined and compared to the other procedures. RF has a 74.8% accuracy rate and a recall of 72.8% which are the greatest among all the algorithms. The complete system design enables the creation of a greater prediction model with better accuracy levels than those achievable with separate categorisation methods. Predicting LOS in the ED through this approach, improvement activities might be implemented to lower its value. First, staffing might be modified depending on the number of patients in the ED to enhance the triage the patient assessment processes. Furthermore, the results obtained might be used to build new protocols for improving ED workflow and regulate the decision-making process for bed utilisation and patient placement based on their severity.

It is not always fair performing a direct comparison with other ML-based studies since the variability between the design of different papers can influence the results. Nevertheless, there have been other researchers who tried to build predictive models to classify ED-LOS.

Turgeman et al. performed a regression analysis by applying a regression tree model for predicting the LOS, based on static inputs (i.e., values that are known at the time of admission and that do not change during patient's hospital stay) (14); they included several tens of predictors and obtained a coefficient of determination between 0.75 and 0.8.

Similarly, Naemi et al. performed a prediction of ED-LOS by using predictors available at the admission including pulse rate, arterial blood oxygen saturation, respiration rate, systolic blood pressure, triage category, arrival ICD-10 codes and gender; they performed both a regression with a final coefficient of determination of 0.33 and a classification with a final accuracy ranging from 66% to 82% (55).

Rahman et al. implemented a decision tree by including 33 attributed to identify patients at high risk of prolonged ED-LOS and reached an accuracy of 85% (56).

In summary, the results of this study can be exploited to develop a preventive plan to optimise the management of EDs by controlling ED-LOS, thus improving ED crowding and the consequent financial costs associated with it. It is undeniable that ED disorganisation causes congestion and delays, as well as influencing decisions of patients to leave the ED before seeing a doctor. This is likely because as the number of patients grows, i.e., during a specific time slot, healthcare professional staff become gradually insufficient and waiting times rise. By predicting prolonged ED-LOS, decision makers could give more attention to the need for supplementary medical, nurses, support staffing in specific work shifts that are well known to be critical. Even though the impacts of the considered parameters may not be universal, the technique might be applied in all the EDs for localised LOS study, with changes planned based on individual observations.

Of course, this study has some limitations; among them, it should be noted that, despite the large number of available data, this is a single-centre study, and the output was made binary. Of note, to implement our workflow, we employed only a few features, which means that the algorithm has been able to achieve such results only by using as input age, gender, triage level, time of admission, and arrival mode. To further differentiate the algorithms’ performance, it would be useful, as a future development, including other features that could be useful to further boost the evaluation metrics. Future improvements of this research might consider important root causes and the access to information, such as shortage of beds, staff shifts, delays in radiology and laboratory units, which would allow us to obtain a more powerful model. Because some of these organisational causes are external and not directly due to the ED process, measures implemented considering also these aspects might cover and improve the overall healthcare chain instead of the ED service only.

Moreover, making a comparison between datasets belonging to similar hospitals could be an interesting development aimed at determining with a more global vision the factors that most influence crowding and the increase of ED-LOS.

Another interesting development could be the implementation of this analysis process on data acquired after the COVID-19 pandemic, to compare the results before and after it.

Finally, we recognise that the number of features included in this study, to make the predictions, is limited. This is, of course, a limitation but, at the same time, it makes us consider that expanding them in future works may be useful to further improve the proposed models.

## Data Availability

The Hospital institution has allowed anonymised data to be used for scientific research purposes, but not for public disclosure. We got anonymised data for research purposes after a formal request. Consequently, the dataset is available from the corresponding author.

## References

[1] Asplin BR Magid DJ Rhodes KV Solberg LI Lurie N Camargo CA Jr. A conceptual model of emergency department crowding. Ann Emerg Med. (2003) 42:173–80. 10.1067/mem.2003.302 12883504

[2] Journey Sibbritt D Isbister GK Walker R. Emergency department performance indicators that encompass the patient. Qual Manag Health Care. (2006) 15(1):27–38. 10.1097/00019514-200601000-00004 16456478

[3] Asaro PV Lewis LM Boxerman SB. Emergency department overcrowding: analysis of the factors of renege rate. Acad Emerg Med. (2007) 14:157–62. 10.1197/j.aem.2006.08.011 17185293

[4] Morris ZS Boyle A Beniuk K Robinson S. Emergency department crowding: towards an agenda for evidence based intervention. Emerg Med J. (2012) 29(6):460–6. 10.1136/emj.2010.107078 21653203

[5] Di Somma S Paladino L Vaughan L Lalle I Magrini L Magnanti M. Overcrowding in emergency department: an international issue. Intern Emerg Med. (2015) 10:171–5. 10.1007/s11739-014-1154-8 25446540

[6] Strada A Bravi F Valpiani G Bentivegna R Carradori T. Do health care professionals’ perceptions help to measure the degree of overcrowding in the emergency department? A pilot study in an Italian University Hospital. BMC Emerg Med. (2019) 19:47. 10.1186/s12873-019-0259-9 31455226 PMC6712594

[7] Todisco C. Overcrowding and clinical risk in emergency departments. A model for the reduction in NEDOCS: preliminary results. Acta Biomed. (2015) 86:170–5. 26422432

[8] Hoot NR Aronsky D. Systematic review of emergency department crowding: causes, effects, and solutions. Ann Emerg Med. (2008) 52:126–36. 18433933 10.1016/j.annemergmed.2008.03.014 PMC7340358

[9] Han JH France DJ Levin SR Jones ID Storrow AB Aronsky D. The effect of physician triage on emergency department length of stay. J Emerg Med. (2010) 39(2):227–33. 10.1016/j.jemermed.2008.10.006 19168306

[10] Parker BT Marco C. Emergency department length of stay: accuracy of patient estimates. West J Emerg Med. (2014) 15(2):170–5. 10.5811/westjem.2013.9.15816 24672606 PMC3966453

[11] Yiadom MY Napoli A Granovsky M Parker RB Pilgrim R Pines JM Managing and measuring emergency department care: results of the fourth emergency department benchmarking definitions summit. Acad Emerg Med. (2020) 27(7):600–11. 10.1111/acem.13978 32248605

[12] Forster AJ Stiell I Wells G Lee AJ van Walraven C. The effect of hospital occupancy on emergency department length of stay and patient disposition. Acad Emerg Med. (2003) 10:127–33. 10.1197/aemj.10.2.127 12574009

[13] Colella Y De Lauri C Maria Ponsiglione A Giglio C Lombardi A Borrelli A Romano M. A comparison of different machine learning algorithms for predicting the length of hospital stay for pediatric patients. *2021 International Symposium on Biomedical Engineering and Computational Biology*; August 13–15, 2021; Nanchang China. Article 39. New York, NY, USA: Association for Computing Machinery. (2021). p. 1–4. 10.1145/3502060.3503648

[14] Turgeman L May JH Sciulli R. Insights from a machine learning model for predicting the hospital length of stay (LOS) at the time of admission. Expert Syst Appl. (2017) 78:376–85. 10.1016/j.eswa.2017.02.023

[15] Scala A Angela Trunfio T Lombardi A Giglio C Borrelli A Triassi M. A comparison of different machine learning algorithms for predicting the length of hospital stay for patients undergoing cataract surgery. *2021 International Symposium on Biomedical Engineering and Computational Biology*; August 13–15, 2021; Nanchang, China. Article 38. New York, NY, USA: Association for Computing Machinery (2021). p. 1–4. 10.1145/3502060.3503647

[16] Bacchi S Tan Y Oakden-Rayner L Jannes J Kleinig T Koblar S. Machine learning in the prediction of medical inpatient length of stay. Intern Med J. (2022) 52(2):176–85. 10.1111/imj.14962 33094899

[17] Ponsiglione AM Trunfio TA Rossi G Borrelli A Romano M. Modelling the length of hospital stay after knee replacement surgery through machine learning and multiple linear regression at “San Giovanni di Dio e Ruggi d'Aragona” university hospital. *2021 10th International Conference on Bioinformatics and Biomedical Science*; October 29–31, 2021; Xiamen, China. New York, NY, USA: Association for Computing Machinery (2021). p. 112–6. 10.1145/3498731.3498748

[18] Daghistani TA Elshawi R Sakr S Ahmed AM Al-Thwayee A Al-Mallah MH. Predictors of in-hospital length of stay among cardiac patients: a machine learning approach. Int J Cardiol. (2019) 288:140–7. 10.1016/j.ijcard.2019.01.046 30685103

[19] Profeta M Maria Ponsiglione A Ponsiglione C Ferrucci G Giglio C Borrell A. Comparison of machine learning algorithms to predict length of hospital stay in patients undergoing heart bypass surgery. *2021 International Symposium on Biomedical Engineering and Computational Biology*; August 13–15, 2021; Nanchang, China. Cham: Springer (2021). p. 518–26.

[20] Ponsiglione AM Cosentino C Cesarelli G Amato F Romano M. A comprehensive review of techniques for processing and analyzing fetal heart rate signals. Sensors. (2021) 21(18):6136. 10.3390/s21186136 34577342 PMC8469481

[21] Benbelkacem S Kadri F Atmani B Chaabane S. Machine learning for emergency department management. Int J Info Sys Serv Sec. (2019) 11(3):19–36. 10.4018/IJISSS.2019070102

[22] Ponsiglione AM Cesarelli G Amato F Romano M. Optimization of an artificial neural network to study accelerations of fetal heart rhythm. In: 2021 IEEE 6th International Forum on Research and Technology for Society and Industry (RTSI;: 06–09 September 2021. Naples Italy: IEEE (2021). p. 159–64. 10.1109/RTSI50628.2021.9597213

[23] Tuominen J Lomio F Oksala N Palomäki A Peltonen J Huttunen H Forecasting daily emergency department arrivals using high-dimensional multivariate data: a feature selection approach. BMC Med Inform Decis Mak. (2022) 22(1):134. 10.1186/s12911-022-01878-7 35581648 PMC9112570

[24] Liu N Chee ML Foo MZQ Pong JZ Guo D Koh ZX Heart rate n-variability (HRnV) measures for prediction of mortality in sepsis patients presenting at the emergency department. PLoS One. (2021) 16(8):e0249868. 34460853 10.1371/journal.pone.0249868 PMC8405012

[25] Shuzan MNI Chowdhury MH Hossain MS Chowdhury ME Reaz MBI Uddin MM A novel non-invasive estimation of respiration rate from motion corrupted photoplethysmograph signal using machine learning model. IEEE Access. (2021) 9:96775–90. 10.1109/ACCESS.2021.3095380

[26] Urteaga J Aramendi E Elola A Irusta U Idris A. A machine learning model for the prognosis of pulseless electrical activity during out-of-hospital cardiac arrest. Entropy. (2021) 23(7):847. 10.3390/e23070847 34209405 PMC8307658

[27] Jiang H Mao H Lu H Lin P Garry W Lu H Machine learning-based models to support decision-making in emergency department triage for patients with suspected cardiovascular disease. Int J Med Inf. (2021) 145:104326. 10.1016/j.ijmedinf.2020.104326 33197878

[28] Liu N Xie F Siddiqui FJ Ho AFW Chakraborty B Nadarajan GD Leveraging large-scale electronic health records and interpretable machine learning for clinical decision making at the emergency department: protocol for system development and validation. JMIR Res Protoc. (2022) 11(3):e34201. 10.2196/34201 35333179 PMC9492092

[29] Ponsiglione AM Marino MR Raiola E Smeraglia F Festa E Russo G Scala A. Analyzing LOS variation for patients under emergency interventions: a bicentric study. *International Symposium on Biomedical and Computational Biology*; August 13–15, 2022; Nanchang China. Cham: Springer International Publishing (2022). p. 453–62.

[30] Fiorillo A Sorrentino A Scala A Abbate V Dell'aversana Orabona G. Improving performance of the hospitalization process by applying the principles of lean thinking. TQM J. (2021) 33(7):253–71. 10.1108/TQM-09-2020-0207

[31] Hofer KD Saurenmann RK. Parameters affecting length of stay in a pediatric emergency department: a retrospective observational study. Eur J Pediatr. (2017) 176:591–8. 10.1007/s00431-017-2879-y 28275860

[32] Stephens RJ White SE Cudnik M Patterson ES. Factors associated with longer length of stay for mental health emergency department patients. J Emerg Med. (2014) 47:412–9. 10.1016/j.jemermed.2014.04.040 25074781

[33] Yoon P Steiner I Reinhardt G. Analysis of factors influencing length of stay in the emergency department. CJEM. (2003) 5(3):155–61. 10.1017/S1481803500006539 17472779

[34] Chan TC Killeen JP Kelly D Guss DA. Impact of rapid entry and accelerated care at triage on reducing emergency department patient wait times, lengths of stay, and rate of left without being seen. Ann Emerg Med. (2005) 46:491–7. 10.1016/j.annemergmed.2005.06.013 16308060

[35] Casalino E Wargon M Peroziello A Choquet C Leroy C Beaune S Predictive factors for longer length of stay in an emergency department: a prospective multicentre study evaluating the impact of age, patient’s clinical acuity and complexity, and care pathways. Emerg Med J. (2014) 31:361–8. 10.1136/emermed-2012-202155 23449890

[36] McHugh M Van Dyke K McClelland M Moss D. *Improving Patient Flow and Reducing Emergency Department Crowding: A Guide for Hospitals*. AHRQ Publication 11(12)-0094. Rockville, MD: Agency for Healthcare Research and Quality (2011).

[37] Adriaenssens J De Gucht V Maes S. Determinants and prevalence of burnout in emergency nurses: a systematic review of 25 years of research. Int J Nurs Stud. (2015) 52(2):649–61. 10.1016/j.ijnurstu.2014.11.004 25468279

[38] Chalfin DB Trzeciak S Likourezos A Baumann BM Dellinger RP, DELAY-ED Study Group. Impact of delayed transfer of critically ill patients from the emergency department to the intensive care unit. Crit Care Med. (2007) 35:1477–83. 10.1097/01.CCM.0000266585.74905.5A 17440421

[39] Henneman PL Nathanson BH Li H Smithline HA Blank FSJ Santoro JP Emergency department patients who stay more than 6 h contribute to crowding. J Emerg Med. (2010) 39:105–12. 10.1016/j.jemermed.2008.08.018 19157757

[40] Ahmed AA Ibro SA Melkamu G Seid SS Tesfaye T. Length of stay in the emergency department and its associated factors at Jimma Medical Center, Southwest Ethiopia. Open Access Emerg Med. (2020) 12:227–35. 10.2147/OAEM.S254239 33116958 PMC7553249

[41] Rose L Scales DC Atzema C Burns KEA Gray S Doing C Emergency department length of stay for critical care admissions. A population-based study. Ann Am Thorac Soc. (2016) 13:1324–32. 10.1513/AnnalsATS.201511-773OC 27111127

[42] McCarthy ML Zeger SL Ding R Levin SR Desmond JS Lee J Crowding delays treatment and lengthens emergency department length of stay, even among high-acuity patients. Ann Emerg Med. (2009) 54:492–503.e4. 10.1016/j.annemergmed.2009.03.006 19423188

[43] Megalla M Ogedegbe C Sanders AM Cox N DiSanto T Johnson H Factors associated with repeat emergency department visits for low back pain. Cureus. (2022) 14:2. 10.7759/cureus.21906 PMC8898564 35265428

[44] Sulis E Di Leva A. An agent-based model of a business process: the use case of a hospital emergency department. *International Conference on Business Process Management*; September 10–15, 2017; Barcelona, Spain. Cham: Springer (2017). p. 124–132.

[45] Rodi SW Grau MV Orsini CM. Evaluation of a fast track unit: alignment of resources and demand results in improved satisfaction and decreased length of stay for emergency department patients. Qual Manag Health Care. (2006) 15:163–70. 10.1097/00019514-200607000-00006 16849988

[46] Pham JC Ho GK Hill PM McCarthy ML Pronovost PJ. National study of patient, visit, and hospital characteristics associated with leaving an emergency department without being seen: predicting LWBS. Acad Emerg Med. (2009) 16:949–55. 10.1111/j.1553-2712.2009.00515.x 19799570

[47] Combes C Kadri F Chaabane S. Predicting hospital length of stay using regression models: application to emergency department. *10ème Conférence Francophone de Modélisation, Optimisation et Simulation-MOSIM'14*; Nov 2014; Nancy, France. Hal Science Ouverte (2014).

[48] Azari A Janeja VP Levin S. Imbalanced learning to predict long stay emergency department patients. *IEEE International Conference on Bioinformatics and Biomedicine, BIBM 2015*; 09–12 November 2015; Washington, DC, USA. Institute of Electrical and Electronics Engineers Inc. (2015).

[49] Wrenn J Jones I Lanaghan K Congdon CB Aronsky D. Estimating patient's length of stay in the emergency department with an artificial neural network. *AMIA Annual Symposium Proceedings*; October 22, 2005 - October 26; Washington, DC, (2005). p. 1155. PMC1560706 16779441

[50] Gul M Guneri AF. Forecasting patient length of stay in an emergency department by artificial neural networks. J Aeronautics Space Technol. (2015) 8(2):43–8.

[51] Giglio C De Lauri C Della Vecchia A Borrelli A Russo G Triassi M Investigation of factors increasing waiting times in the emergency departments of “San Giovanni di Dio e Ruggi d'Aragona” hospital through machine learning. In 2021 International Symposium on Biomedical Engineering and Computational Biology (2021). p. 1–5.

[52] Driesen BE Van Riet BH Verkerk L Bonjer HJ Merten H Nanayakkara PW. Long length of stay at the emergency department is mostly caused by organisational factors outside the influence of the emergency department: a root cause analysis. PLoS One. (2018) 13(9):e0202751. 10.1371/journal.pone.0202751 30216348 PMC6138369

[53] van der Veen D Remeijer C Fogteloo AJ Heringhaus C de Groot B. Independent determinants of prolonged emergency department length of stay in a tertiary care centre: a prospective cohort study. Scand J Trauma Resusc Emerg Med. (2018) 26(1):1–9. 10.1186/s13049-018-0547-5 30236125 PMC6148782

[54] Raschka S. Model evaluation, model selection, and algorithm selection in machine learning. arXiv preprint arXiv:1811.12808 (2018).

[55] Naemi A Schmidt T Mansourvar M Ebrahimi A Wiil UK. Quantifying the impact of addressing data challenges in prediction of length of stay. BMC Med Inform Decis Mak. (2021) 21:1–13. 10.1186/s12911-021-01660-1 34749708 PMC8576901

[56] Rahman MA Honan B Glanville T Hough P Walker K. Using data mining to predict emergency department length of stay greater than 4 h: derivation and single-site validation of a decision tree algorithm. Emerg Med Australas. (2020) 32(3):416–21. 10.1111/1742-6723.13421 31808312

